# Placental Malaria is associated with reduced early life weight development of affected children independent of low birth weight

**DOI:** 10.1186/1475-2875-9-16

**Published:** 2010-01-14

**Authors:** Brigitte Walther, David JC Miles, Sarah Crozier, Pauline Waight, Melba S Palmero, Olubukola Ojuola, Ebrima Touray, Marianne van der Sande, Hilton Whittle, Sarah Rowland-Jones, Katie L Flanagan

**Affiliations:** 1 MRC Laboratories, Atlantic Boulevard, Fajara, PO Box 273 Banjul, The Gambia; 2 South African Tuberculosis Vaccine Initiative, Institute of Infectious Diseases and Molecular Medicine, and School of Child and Adolescent Health, University of Cape Town, Observatory, South Africa; 3 MRC Epidemiology Resource Centre, University of Southampton, Southampton General Hospital, Southampton, UK; 4 Immunization Department, Health Protection Agency Centre for Infections, Colindale Avenue, London, UK; 5 Adventist Community Services of Alaska, 6100 O'Malley Road, Anchorage, USA; 6 Department of Pediatrics, Bronx-Lebanon Hospital Center, 1650 Grand Concourse, Bronx, New York, USA; 7 Epidemiology and Surveillance Unit, Centre for Infectious Diseases Control, National Institute for Public Health and the Environment, Bilthoven, Netherlands; 8 MRC Human Immunology Unit, Weatherall Institute of Molecular Medicine, John Radcliffe Hospital, Oxford, UK

## Abstract

**Background:**

Infection with *Plasmodium falciparum *during pregnancy contributes substantially to the disease burden in both mothers and offspring. Placental malaria may lead to intrauterine growth restriction or preterm delivery resulting in low birth weight (LBW), which, in general, is associated with increased infant morbidity and mortality. However, little is known about the possible direct impact of the specific disease processes occurring in PM on longer term outcomes such as subsequent retarded growth development independent of LBW.

**Methods:**

In an existing West-African cohort, 783 healthy infants with a birth weight of at least 2,000 g were followed up during their first year of life. The aim of the study was to investigate if *Plasmodium falciparum *infection of the placenta, assessed by placental histology, has an impact on several anthropometric parameters, measured at birth and after three, six and 12 months using generalized estimating equations models adjusting for moderate low birth weight.

**Results:**

Independent of LBW, first to third born infants who were exposed to either past, chronic or acute placental malaria during pregnancy had significantly lower weight-for-age (-0.43, 95% CI: -0.80;-0.07), weight-for-length (-0.47, 95% CI: -0.84; -0.10) and BMI-for-age z-scores (-0.57, 95% CI: -0.84; -0.10) compared to infants born to mothers who were not diagnosed with placental malaria (p = 0.019, 0.013, and 0.012, respectively). Interestingly, the longitudinal data on histology-based diagnosis of PM also document a sharp decline of PM prevalence in the Sukuta cohort from 16.5% in 2002 to 5.4% in 2004.

**Conclusions:**

It was demonstrated that PM has a negative impact on the infant's subsequent weight development that is independent of LBW, suggesting that the longer term effects of PM have been underestimated, even in areas where malaria transmission is declining.

## Background

Despite the availability of appropriate tools to control malaria and recently strengthened efforts to employ them efficiently [1], the latest estimates still consider 3.3 billion people at risk of acquiring malaria each year. In 2006, 247 million malaria cases were reported, causing almost one million deaths [2]. Approximately 50 million pregnant women are at risk of malaria, more than 50% of whom live in areas of sub-Saharan Africa with intense transmission of *Plasmodium falciparum*[3]. In these areas at least one in four pregnant women (all parities) has evidence of malaria infection at delivery defined as either peripheral malaria or placental malaria (PM) [4,5]. Where transmission is low, peripheral and placental parasitaemia prevalence was estimated as 13.7% and 6.7%, respectively [6].

The deleterious effects of malaria in pregnancy on the morbidity and mortality of fetuses and newborns are well recognized. The impact of PM on the mother depends on the level of acquired immunity and is more severe in non-immune women. In highly endemic areas, where women can be assumed to be partially immune, the frequency and severity of the infection is highest in primigravidae. The main effects of malaria in pregnancy on birth outcomes are thought to be mediated by maternal anaemia [7,8] and placental insufficiency [9-13]. Both factors have been suggested to act together to cause either intrauterine growth restriction (IUGR) [14] or preterm delivery [15,16], leading to low birth weight (LBW) [17-19]. A number of studies have demonstrated a firm association between PM and LBW [4,5,13,20], and in general, LBW itself is known to be highly associated with infant morbidity and mortality [21,22]. Likewise, in children born to mothers with PM, the associated LBW has been suggested as a major risk factor for neonatal and infant mortality [23-25]. It has been estimated that PM causes 167,000-967,000 cases of LBW in Africa, associated with 62,000-363,000 newborn deaths each year [4,26]. However, it has been emphasized that these infant deaths are not due to the impact of LBW alone, but to the general disease processes which have resulted in LBW [27]. In addition, PM has been associated with a twofold increase in risk of stillbirth [28], and may put the foetus at increased risk of congenital cytomogalovirus infections [29]. While the immediate consequences of PM and LBW on neonatal and infant morbidity and mortality have been studied extensively, comparatively little is known about the possible long-term impact of PM on the subsequent development of the child. To date only one study has shown that 12 month old infants exposed to maternal malaria at delivery (placental or peripheral) were lighter and thinner than children who were not exposed to PM [30], even after controlling for low birth weight.

Since LBW in itself, regardless of the underlying processes, has been associated with inhibited growth, impaired cognitive development, and increased incidence of chronic diseases later in life [31], it is difficult to study potential direct effects of PM on the subsequent development of the child that are independent of the effects caused by the associated LBW. This study addressed whether PM has an additional or 'independent' effect on growth development, apart from the known effects of PM following the causal pathway (exposure to PM → LBW → incomplete catch-up growth [30] → growth retardation). The direct impact of PM on the child's subsequent growth development during the first year of life was investigated by using data from an ongoing Gambian cohort study, which was specifically designed to enroll healthy babies, excluding those with a birth weight below 2,000 g. This study design and subsequent analysis adjusting for moderate low birth weight (2,000 g - 2,499 g) offered the unique opportunity to study the impact of PM independent of LBW or overt co-morbidity.

## Methods

### Study participants and recruitment

Data were collected from children born between January 2002 and July 2005 as part of an ongoing open cohort study at the maternity ward of the Health Centre in Sukuta, a semi-urban Gambian village 30 km south of the capital Banjul, where malaria transmission varies considerably between season, with highest incidence during the wet season and immediately afterwards ('malaria season': August - December). The main study, as well as the analysis strategy of the data presented here, was approved by the Joint Gambian Government/MRC Ethics Committee. The newborns delivered at the maternity ward were enrolled after informed consent of the mother was obtained. The purpose of the main cohort was to study immune responses of infants to vaccines and infections. Since overt morbidity, as well as risk factors associated with LBW, are known to increase susceptibility to infectious diseases and thus bias any immunological responses [32-35] only healthy singleton children were eligible for recruitment into the cohort. Newborns below a birth weight of 2,000 g were excluded from the study.

A survey among Gambian pregnant women in 2000-01 [36] estimated the prevalence of HIV to be 1.0% (CI: 0.9-2.4%) in Serekunda, an urban settlement approximately 10 km from Sukuta. Based on this estimate, HIV+ cases are unlikely to have significantly impacted on the results of this study, and following ethical guidance HIV testing was not performed.

### Study Design

Demographic, anthropometric and clinical data from 783 mother/child pairs were used to investigate the association of maternal PM and height and weight in the first year of life of the offspring. Basic demographic and socio-economic data, such as, ethnicity, age, and parity of the mother, education of the parents and, as a measure for overcrowding, number of persons sleeping in one bedroom, as well as information on malaria treatment and bednet use were collected in face-to-face interviews by trained fieldworkers using questionnaires shortly after birth. Infants were followed up monthly over a 12-month period for morbidity and anthropometric measurements. At each follow up visit trained nurses recorded the number of illness episodes during the previous month reported by the mother or guardian of the child and administered vaccines according to the recommended Gambian Expanded Programme of Immunization schedule. Maternal height and weight were recorded six months after delivery.

PM status was defined by placental histology. After birth a placental biopsy was taken and embedded in paraffin and stained for histological analysis of PM status [29]. Cases were classified according to Ismail [37] as active infection (presence of infected red blood cells in the intervillous space of the placenta) and past infection (no parasites, but haemozoin deposition in macrophages). Active infection was further subdivided into acute infections (only parasites and minimal haemozoin deposition) and chronic infections (parasites and significant haemozoin deposition).

Childrens' weight and length measurements at three, six and 12 months of age (+/- 15 days) were used to calculate four age/gender-standardized indicators: 'weight-for-age' (wfa), 'length-for-age' (lfa), 'weight-for-length' (wfl) and 'body-mass-index-for-age' (BMIfa) based on the WHO "Child Growth Standards, 2006" using the Stata macro 'igrowup.ado'[38].

### Statistical methods

All data were double entered into an Access database (Microsoft), validated and checked for range and consistency. Prior to investigating if PM is associated with infant's size and nutritional status, univariable analyses were used to assess whether known PM risk factors (young maternal age, low parity, low socio-economic status, no bednet use, and seasonality of malaria transmission) [6] were present in this study population. A logistic regression model was fitted including all risk factors with p-values less than 0.2 in univariable analysis, which were kept in the model at a significance level p ≤ 0.05. The final model including parity, PM season and year, and duration of schooling of the mother was used to calculate adjusted ORs and corresponding p-values of being PM+ comparing the individual exposure groups to a specified baseline group.

The hypothesis that exposure to PM would be associated with lower values for anthropometric indicators was tested at each of the three time points three, six and 12 months using univariable analyses first. Additional mother and child characteristics such as maternal age, height, BMI, parity, duration of schooling, living in crowded housing conditions as well as gender of the child, moderate low birth weight of 2,000 g - 2,499 g, current age of the child, month and year of birth, and whether the child was born in the 'hungry' season (during the months with intensive rain fall from July to October) [39,40] were investigated as risk factors for reduced indicators. Univariable analyses were conducted to examine the association of each factor with the four anthropometric indicators described above at three, six and 12 months of age.

Cross-sectional times series models using generalized estimating equations (GEE) to fit the parameters of the models with exchangeable correlation structure were then constructed for each anthropometric indicator. The final models account for multiple weight and length measurements at three, six and 12 months of age and confounding factors such as sex, moderate low birth weight of 2,000 g - 2.499 g, infant's age, birth month and year, parity and education (duration of schooling) of the mother. An interaction term was included for PM exposure status of the child and parity to investigate if the association of PM and size of the infant varied between parity groups. Since the association of PM and the growth indicators wfa, wfl and BMIfa was not significantly different for two or three pregnancies, but for more than four pregnancies compared to the first pregnancy, data was split into two groups according to 1-3 and 4 and more pregnancies. Maternal age and living in crowded housing conditions lacked a significant association with z-scores and was therefore not included in the final models. Mother's height and BMI were not considered, since 61% of the data were missing. No difference was seen for anthropometric measurements taken during or after the 'hungry' season; thus "hungry season" was not included in the models. The resulting models were used to assess the effect of PM exposure on each anthropometric indicator independent of birth weight.

Whether maternal exposure to PM was associated with being underweight, wasted or stunted (wfa, wfl or lfa z-scores<-2, respectively) was investigated using logistic regression. Odds ratios for the group of wasted or underweight infants and a second group of stunted infants were calculated choosing all the remaining well-nourished infants as the baseline group.

## Results

### Baseline characteristics of the study population

Available data from 783 mother/child pairs, collected between January 2002 and July 2005 were analysed. In each year, with the exception of 2005, a comparable number of babies were enrolled and genders were equally distributed with 54.2% (SD 0.5%) being male. Mean birth weight and length were 3,045 g (SD 449 g) and 48.5 cm (SD 2.2 cm), respectively. 80 babies (10.3%) had a birth weight of 2,000 g - 2,499 g. Mean maternal age was 25.6 years (SD 5.5 years). 74 (9.5%) placentas were *P. falciparum *infected; 37/74 (50%) of which were classified as active infection, which was further sub-divided into 21/74 (28.4%) acute and 16/74 (21.6%) chronic infections. The remaining 50% were classified as past infected. Further maternal and child characteristics are summarized in Table 1.

**Table 1:** Baseline characteristics of the study population for 2002-05

**Maternal characteristics**, N = 783	**No**	**(%)**
**Age of the mother**		
Age <20	99	(12.6)
Age 20-24	252	(32.2)
Age 25-29	219	(28.0)
Age ≥30	179	(22.9)
Unknown	34	(4.3)
**Number of pregnancies**		
First	153	(19.5)
Second	173	(22.1)
Third	123	(15.7)
Fourth	100	(12.8)
Fifth	81	(10.4)
Sixth or more	153	(19.5)
**Mother's ethnic group**		
Mandinka	426	(54.4)
Fula	95	(12.1)
Wolof	87	(11.1)
Jola	78	(10.0)
Serere	37	(4.7)
Serahula	22	(2.8)
Manjago	11	(1.4)
Other	27	(3.5)
**Duration of education of the mother**		
0-4 years	199	(25.4)
5 and more years	403	(51.5)
Unknown	181	(23.1)
**Placental malaria**		
Not infected	709	(90.6)
Acute	21	(2.7)
Chronic	16	(2.0)
Past	37	(4.7)
**Baby's characteristics**, N = 783		
**Year of birth**		
2002	164	(21.0)
2003	218	(27.8)
2004	277	(35.4)
2005 (January - July)	124	(15.8)

### Factors associated with PM

The cohort was assessed for known risk factors for PM [6]. As has been described previously, PM was significantly associated with first pregnancy and lower socio-economic status of the mother. However, in this cohort initial univariable analysis did not reveal a significant association of maternal age, BMI, ethnicity, treatment for malaria and bednet use with PM (Additional file 1).

In the presented study, maternal anaemia, a consequence of PM known to be associated with LBW [13,17,41-44] was not associated with PM and newborns in the PM+ group were not significantly lighter than newborns in the PM- group (Additional file 2). During the one year follow up infants in both comparison groups suffered a similar number of all-cause morbidity episodes.

### Lower z-scores for anthropometric indicators for children born to PM+ mothers independent of LBW

To assess the impact of PM status at birth on growth development during the first year of life, 1,491 (PM+: 137, PM-: 1354) z-scores for each of four anthropometric indicators (wfa, lfa, BMIfa, and wfl) were analysed (528 at three months, 510 at six months and 453 at 12 months of age). At all time points mean z-scores for wfa, lfa, and BMIfa were significantly lower than zero (mean range across age: wfa: -0.59 to -0.26; lfa: -0.67 to -0.27; and BMIfa: -0.29 to -0.10), indicating that Gambian children recruited in the cohort were lighter, smaller, and had lower values for BMI than children of the world standard population with same sex and age characteristics (p < 0.0001). Mean wfl z-scores were significantly lower than zero at six months (-0.05, p = 0.038) and 12 months (-0.36, p < 0.0001), but not at three months (0.06, p = 0.565).

**Figure 1:**
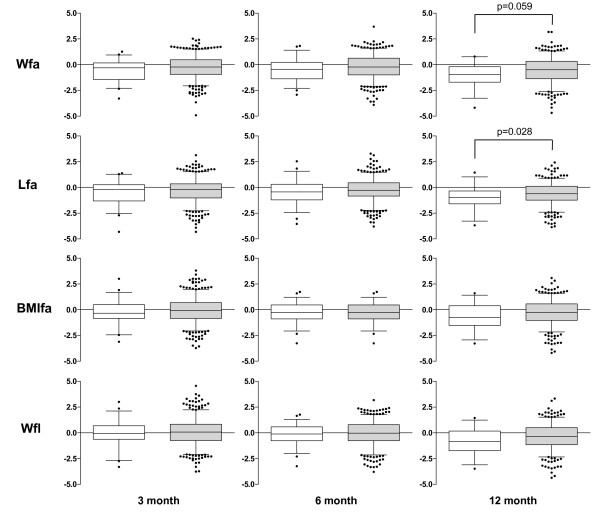
**Anthropometric indicators of infants who were either placental malaria exposed or unexposed during pregnancy**. Median, inter quartile range, and range (minimum to maximum value) of four anthropometric indicators weight-for-age, length-for-age, BMI-for-age and weight-for-length are presented for infants born to placental malaria positive (open boxes) and placental malaria negative mothers (black boxes) at the age of three, six, and 12 months.

Using univariable analysis comparing children born to PM+ mothers with those born to PM- mothers, infants exposed to PM had at least borderline significantly lower mean z-scores for all four anthropometric indicators at 12 months (Figure 1). Mean differences ranged from 0.29 z-scores (CI: 0.18; 0.40) to 0.60 z-scores (CI: 0.48; 0.70). The mean differences in z-scores between 12 and three months were not significantly different between PM+ and PM- groups (wfa: p = 0.738, lfa: p = 0.790, BMIfa: p = 0.9995, and wfl: p = 0.668).

Longitudinal analysis using generalized estimating equations (GEE) accounting for multiple measurements for infants at different time points, and for risk factors for low anthropometric indicators such as parity and duration of maternal education, moderate low birth weight (2,000 g - 2.499 g), gender, month and year of birth, revealed that wfa, BMIfa and wfl z-scores were significantly associated with maternal PM status for first to third borns. However, PM exposure had no effect on lfa z-scores (Table 2).

**Table 2:** Association of placental malaria* and anthropometric indicators of infants

Anthropometric indicator	z-scores difference [(PM+) - (PM-)]**	CI 95%	p-value
**Weight-for-age**			

1-3 pregnancies	-0.43	-0.80; -0.07	0.019

≥4 pregnancies	0.02	-0.44; 0.48	0.930

**Weight-for-length**			

1-3 pregnancies	-0.47	-0.84; -0.10	0.013

≥4 pregnancies	0.03	-0.44; 0.50	0.903

**BMI-for-age**			

1-3 pregnancies	-0.57	-0.84; -0.10	0.012

≥4 pregnancies	0.03	-0.43; 0.50	0.889

**Length-for-age**			

All parities	-0.11	-0.37; 0.15	0.405

* Total of past, chronic and active placental malaria (PM)

**Difference in z-scores of PM exposed and unexposed infants (z-score (PM+) - z-score (PM-))

The table presents the results of the Generalized Estimating Equations models investigating a possible association of exposure to maternal PM during pregnancy and anthropometric indicators (weight-for-age (wfa), weight-for-length (wfl), BMI-for-age (BMIfa) and length-for-age (lfa)) of the infants. 1,488 (PM+:135, PM-:1353) z-scores for each of four anthropometric indicators were included in the GEE model (1-3 pregnancies: 833 observations (PM+:86, PM-:747); ≥4 pregnancies: 655 observations (PM+: 49, PM-: 606)). The models account for multiple weight and length measurements at three, six and 12 months of age and confounding factors such as moderate low birth weight below (2,000 g - 2,499 g), infant's age, sex, birth month and year, and duration of maternal education. Significant interactions between PM infection and parity were fitted for the first three anthropometric indicators (wfa, wfl, and BMIfa), but not for lfa.

Figure 1, representing the raw data for each of the four anthropometric indicators at the three time points for PM+ and PM- groups, implies that the impact of PM on anthropometric indicators might be stronger within the third age group than within the first two groups. The tests for an interaction of PM and age for the final GEE models adjusting for confounding factors such as gender, duration of maternal education and parity, moderate low birth weight, birth month and year of the infant were not significant. The presented data do therefore not support the hypothesis of an effect gradation with increasing age.

At the age of three, six and 12 months 20.1%, 9.6%, and 22.2% of the infants in the PM+ group were either wasted, underweight or stunted compared to 14.2%, 13.1%, and 17.0% in the PM- group, respectively. The odds for wasting or being underweight were 2.4 and 3.1 times higher in the PM exposed group at the age of three and 12 months respectively, but did not reach significance (p = 0.095 for both). Infants of the PM+ groups were not significantly more likely to be stunted compared to infants of the PM- group within any age group.

### Steady decline in PM prevalence from January 2002 to July 2005

**Figure 2:**
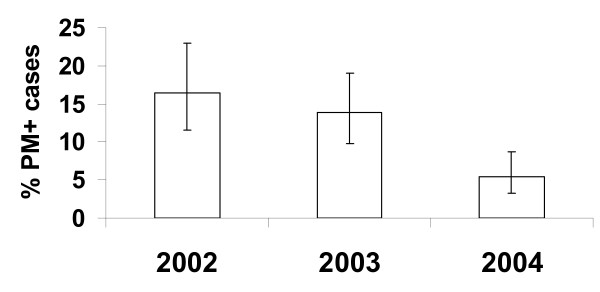
**Decline in placental malaria prevalence during 2002-2004**. The percentage of *P. falciparum *infected placentas (assessed by histology) among all women giving birth is shown per annum, p_trend _< 0.0001.

As shown in Figure 2, the percentage of mothers with PM seen at the Sukuta Health Centre decreased significantly from 16.5% (27/164) in 2002, to 5.4% (15/277) in 2004 (p_trend _< 0.0001). For 2005, data were available for the non transmission season only (January to July), but the risk of PM during the non transmission season was significantly lower in 2004 and 2005 than in 2002 (odds ratio (OR)_2004 _0.42, CI (0.18; 0.95), p = 0.038, OR_2005 _0.08, CI (0.02; 0.37), p-value = 0.001, respectively), with a highly significant trend (p_trend _< 0.001).

### Influence of seasonal malaria transmission on PM prevalence

**Figure 3:**
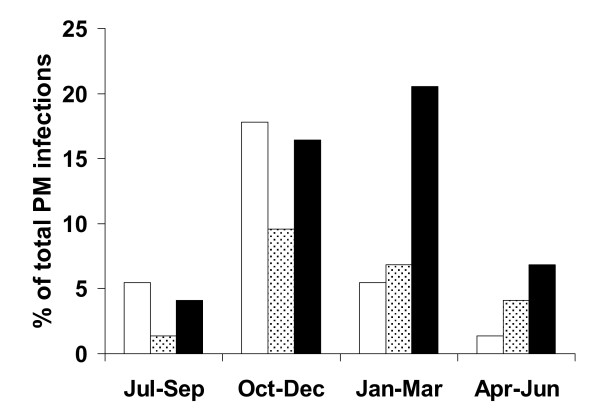
**Seasonality of placental malaria**. The percentage of acute (open bars), chronic (dotted bars) and past (black bars) placental malaria infections is shown over time. From January 2002 till December 2004 21/72 (29.2%) were classified as acute, 16/72 (22.2%) as chronic, and 35/72 (48.6%) as past infection.

The majority of PM cases (55/74, 76%) were diagnosed during the months of October to March (OR 2.85, CI (1.62; 5.03), p < 0.0001). Mothers delivering during the malaria transmission period (August to December) were more likely to be diagnosed with acute PM compared to mothers delivering during the non-transmission period from January to July (OR 3.00, CI (1.16; 7.79), p = 0.024; p_trend _< 0.011 [AOR 3.00, CI (1.14; 7.94), p* = 0.026, p_trend _= 0.014]). By contrast, seasonality of malaria transmission had no effect on chronic infection prevalence (OR 1.12, CI (0.42; 3.04), p = 0.82). A delayed seasonal effect could be observed for past infections in that they were significantly more likely to be diagnosed in the last three months of the malaria transmission season or thereafter through to March of the following year (OR 2.97, CI (1.33; 6.66), p < 0.008; [adjusted OR (AOR) 3.97, CI (1.67; 9.40), adjusted p-value (p*) = 0.005]) (Figure 3).

## Discussion

By taking advantage of a cohort study specifically designed to recruit healthy babies, and subsequent analysis adjusting for moderate low birth weight, the direct impact of PM on the infant's size during the first year of life independent from LBW-associated effects was examined. The main aim of this study was to investigate if babies who were exposed to PM showed a significantly different growth pattern during infancy compared to babies who were not exposed to PM, when the babies in both groups had a similar weight distribution at birth. The finding that the birth weights of the PM+ and PM- groups were not significantly different suggests that we successfully enrolled babies of similar birth weight distribution in both groups. Therefore any PM effects on subsequent growth could not be accounted for by a lower starting birth weight in the PM+ group.

This study shows that first to third born infants of PM+ mothers were significantly lighter, thinner and had lower BMIfa z-scores compared to infants of PM- mothers. The analysis comprised all PM exposed infants disregarding the histological subgroup of PM. Since severity of pathological changes within the placenta, gestational age at infection or duration of infection are closely associated with PM subgroups, it is likely that the association of growth development might be different within the subgroups past, chronic and active PM. Unfortunately this study wasn't sufficiently powered to enable performance of such subgroup analyses.

PM exposed infants were 2.4 times more likely to be wasted at the age of three months and 3.1 times more likely to be underweight at the age of 12 months than infants born to PM- mothers. Since the described effect is independent of LBW, this suggests that the overall burden of PM may have been underestimated. In addition, it should be borne in mind that irrespective of PM status, and despite the exclusion of babies with low birth weight (≤ 2,000 g), infants recruited in the Sukuta cohort were significantly lighter, smaller, and had lower values for BMI than children of the world standard population. Any additional negative effect on nutritional status such as that we described here may therefore become even more relevant for these children.

Overall infants had significantly lower z-scores at 12 months compared to z-scores at three month of age, an effect which might be explained by a change in feeding practice and increased mobility of the infant and therefore higher exposure to pathogens. A limitation of the study is that information on gestational age and breast feeding practices, an important correlate of postnatal growth, was not collected. However, most mothers exclusively breast feed for 6 months, and thereafter continue breast feeding alongside giving solid food past the age of 1 year. The possibility that the described association of PM and infant's growth development has been confounded by maternal height and BMI cannot be excluded. However, among those mothers with available data for height and BMI (39%), median values were not significantly different between PM+ and PM- mothers.

The presented findings imply that the increased risk for poorer weight gain independent of LBW applies to the first to third born infants of PM+ mothers, but not for ≥ fourth born children. Gravidity effects have been described in studies analyzing for the effect of PM on subsequent susceptibility to malaria in early life [34,45]. These gravidity effects have been attributed to the higher malaria antigen load and greater degree of placental inflammation that is thought to occur in the lower gravidity states, since these woman have lower anti-chondroitin sulfate A antibody levels and thus less immunity against PM [45]. However gravidity related altered susceptibility to malaria itself is unlikely to account for the delayed growth in the PM exposed group in this study, since diagnosed malaria was rare in the 1^st ^year of life in this population. Therefore other immunological mechanisms need to be considered.

The inflammatory effects of PM are generally thought to bias the neonatal immune system towards a Th1 inflammatory response [46], and this immune bias could lead to more severe inflammation in response to other infectious diseases encountered in the 1^st ^year of life. Several groups have proposed that immunological tolerance or regulatory T cells might also be primed by PM, which might then result in altered susceptibility to malaria, and also have a non-specific immunosuppressive role [47,48]. Indeed, several studies have since shown that malaria-specific Tregs are primed by PM [49-52]. It has also been shown that non-specific Tregs can be induced by PM, possibly due to priming of innate responses by malaria antigens via pattern recognition receptors such as the toll like receptors [52]. Indeed, TLR mutations have been shown to play an important role in the outcome of pregnancy associated malaria, and in particular TLR-4 and -9 mutations are associated with increased risk of low birth weight in term infants [53]. Altered innate immunity in early life and increased non-specific Tregs could certainly enhance susceptibility to other pathogens and antigens and therefore result in poorer growth development. The number of all cause morbidity episodes between PM exposed and PM not exposed infants were not significantly different in the presented study, although numbers were small and the severity and duration of illness was not assessed.

One further mechanism as to how PM might influence early life development independent of birth weight is its effects on leptin levels. Leptin regulates appetite and metabolism, and during pregnancy the placenta acts as an additional source of leptin [54]. PM has been shown to cause a disruption in the relationship between leptin and birth weight [55], and although it is not known whether leptin responsiveness is later restored, an early lack of leptin responsiveness could certainly contribute to poor growth in PM+ children. Clearly the effect of PM on subsequent immunity in early life is a research an area of great interest, and the precise immunological effects have yet to be fully elucidated.

Several publications report a marked decline of malaria prevalence over the recent years in sub-Saharan Africa [56-60]. For The Gambia, a retrospective record based analysis of malaria incidence spanning from 1999 to 2007 carried out in a region that included the catchment area of Sukuta Health Centre, observed a marked decline in the annual number of admissions and deaths attributed to malaria, particularly since 2003 [61]. Malaria prevalence surveys carried out at the end of the transmission season 2007/8 document unprecedentedly low levels of malaria in the Gambia and Guinea Bissau [62]. The presented data on histology-based diagnosis of PM support this further, documenting a decline in malaria incidence. While in 2002 the study site could have been classified as a high transmission region, based on a PM prevalence of 16.5%, PM prevalence decreased markedly to 5.4% in 2004, indicative of low levels of malaria transmission [6]. Since yearly rainfall data for the Gambia, including the study area Sukuta, do not indicate a significant reduction in average rainfall during the study period, it is rather unlikely that changes in rainfall could account for the observed steep decline in PM prevalence.

A steady increase in bednet coverage in The Gambia for children <5 years from 42% in 2000 to 63% in 2005 [63] may have contributed to this decline, although in this study bednet usage was comparable among PM+ and PM- mothers. Intermittent preventative treatment for malaria in pregnancy with sulphadoxine-pyrimethamine (Fansidar®) was introduced at Sukuta HC antenatal clinic in 2005 and therefore cannot account for the decline in PM.

Earlier reports suggest a delayed seasonal effect on PM prevalence in areas of seasonal transmission [13], which is confirmed by the presented data. Using placental histology it was demonstrated that the vast majority of PM cases detected in the non transmission season are attributable to past placental infections, reaching a peak in January to March. As expected, those cases classified as acute PM closely follow the seasonal transmission pattern, while no significant seasonal variation could be observed in the incidence of chronic PM.

A previous study in a semi-urban area of The Gambia showed socio-economic factors, but not education, to be significantly associated with increased risk for *P. falciparum *infection [64]. In particular, poor and crowded housing conditions were identified as risk factors. While crowding was also associated with PM in the presented dataset, poorer maternal education (duration of schooling) was identified as an additional risk factor for PM. Adequate maternal education may therefore help reducing the burden of PM.

## Conclusions

In summary, it was shown that PM exposure during pregnancy has a negative impact on the infant's growth development for first to third born infants that is independent of, but additional to, the well-documented deleterious effects of LBW associated with PM [4,5,20,23,24,65] This suggests that the burden of PM on the infant's development may have been underestimated, which points out the paramount importance of control strategies for malaria in pregnancy. The main findings of this study, the additional impact of PM on development of infants and the decline in PM over time, illustrate the importance of epidemiological data in designing control measures and determining treatment priorities.

## Competing interests

The authors declare that they have no competing interests.

## Authors' contributions

DJCM, MVDS, HW, SRJ, and KLF contributed to the conception and design of the study. DJCM, PW, MSP, OO, ET, MVDS, and KLF contributed to study implementation and data collection. BW, DJCM, SC performed data analyses and interpreted the data. BW drafted the manuscript and DJCM, SC, MVDS, HW, SRJ, and KLF revised it critically. All authors read and approved the final manuscript.

## Supplementary Material

Additional file 1 **Risk factors for placental malaria for mothers delivering at maternity ward of Sukuta Health Centre from January 2002 to July 2005, multivariable analysis for 2002-04**. The table provided represents the results of the statistical analysis assessing the risk factors for placental malaria (PM). Click here for file

Additional file 2 **Association of placental malaria with maternal anaemia and baby's characteristics at birth**. The table in this file shows the results of the statistical analysis investigating if maternal and baby's outcome characteristics at birth were different between PM+ and PM- groups. Click here for file
