# Supplementary material for: Placental Malaria is associated with reduced early life weight development of affected children independent of low birth weight
Source: Malar J. 2010 Jan 14;9:16. doi: 10.1186/1475-2875-9-16 (PMC2841609; doi:10.1186/1475-2875-9-16)
Supplement: Additional file 1 — Risk factors for placental malaria for mothers delivering at maternity ward of Sukuta Health Centre from January 2002 to July 2005, multivariable analysis for 2002-04. The table provided represents the results of the statistical analysis assessing the risk factors for placental malaria (PM). [file 1475-2875-9-16-S1.DOC]

**Additional file 1:**

**Risk factors for placental malaria for mothers delivering at maternity ward of Sukuta Health Centre from January 2002 to July 2005, multivariable analysis for 2002-04**

|  | **Univariable analysis** | | | | **Multivariable analysis** | | |
| --- | --- | --- | --- | --- | --- | --- | --- |
|  | **2002-05** | | | | **2002-04** | | |
|  | **PM infected** | **Not PM infected** | OR (95% CI) | P value | OR (95% CI) | | P value ; adjusted p value* |
|  |  |  |  |  | Crude | Adjusted* |  |
| **Year of birth,** N=783 | n=74 | n=709 |  |  |  |  | trend <0.0001** |
| 2002 | 27 (36.5) | 137 (19.3) | 1 |  | 1 | 1 | -- |
| 2003 | 30 (40.5) | 188 (26.5) | 0.81 (0.46; 1.42) | 0.464 | 0.81 (0.46; 1.42) | 1.02 (0.56; 1.84)* | 0.464; 0.949* |
| 2004 | 15 (20.3) | 262 (37.0) | 0.29 (0.15; 0.56) | <0.0001 | 0.29 (0.15; 0.56) | 0.29 (0.15; 0.58)* | <0.0001; <0.0001* |
| 2005 (January – July) | 2 (2.7) | 122 (17.2) | *0.08 (0.02; 0.36)**** | *0.001**** | -- | -- | -- |
| **Season for PM infection**  (years 2002-04 only)**,** N=659 | n=72 | n=587 |  |  |  |  |  |
| April-September | 17 (23.6) | 275 (46.9) | -- | -- | 1 | 1 | -- |
| October-March | 55 (76.4) | 312 (53.2) | -- | -- | 2.85 (1.62; 5.03) | 3.06 (1.79; 5.50)* | <0.0001; <0.0001* |
| **Age of the mother,** N=783 | n=74 | n=709 |  |  |  |  |  |
| Age <20 | 10 (13.5) | 89 (12.6) | 0.90 (0.42; 1.93) | 0.784 |  |  |  |
| Age 20-24 | 28 (37.9) | 224 (31.6) | 1 | -- |  |  |  |
| Age 25-29 | 20 (27.0) | 199 (28.1) | 0.80 (0.44; 1.47) | 0.479 |  |  |  |
| Age ≥30 | 12 (16.2) | 167 (23.5) | 0.57 (0.28; 1.17) | 0.121 |  |  |  |
| unknown | 4 (5.4) | 30 (4.2) | 1.07 (0.35; 3.26) | 0.910 |  |  |  |
| Mean age of the mother, N=749 | n=70 | n=679 |  |  |  |  |  |
| Mean (95% CI) | 24.7 (23.6-25.9) | 25.7 (25.3-26.1) |  | 0.163 |  |  |  |
| **Number of pregnancies,** N=783 | n=74 | n=709 |  |  |  |  |  |
| First | 23 (31.0) | 130 (18.3) | 1.95 (1.12; 3.39) | 0.018 | 2.16 (1.22; 3.80) | 2.56 (1.39; 4.72)* | 0.008; 0.003* |
| Second | 13 (51.4) | 160 (22.6) | 0.90 (0.47; 1.73) | 0.742 | 1.10 (0.56; 2.13) | 1.35 (0.67; 2.70)* | 0.789; 0.403* |
| Third or more | 38 (17.6) | 419 (59.1) | 1 | -- | 1 | 1 | -- |
| **BMI of the mother 6 months after birth**, N=308 | n=25 | n=283 |  |  |  |  |  |
| BMI [median (IQR)] | 21.2 (20.6-23.4) | 21.0 (19.0-23.6) |  | 0.253 |  |  |  |
| **Height of the mother** [cm], N=308 |  |  |  |  |  |  |  |
| BMI [median (IQR)] | 160 (157-163) | 161 (157-165) |  | 0.181 |  |  |  |
| **Mother's ethnic group,** N=783 | n=74 | n=709 |  |  |  |  |  |
| Mandinka | 37 (50.0) | 389 (54.9) | 1 | -- |  |  |  |
| Fula | 11 (14.9) | 84 (11.9) | 1.38 (0.67; 2.81) | 0.380 |  |  |  |
| Wolof | 10 (13.5) | 77 (10.9) | 1.37 (0.65; 2.86) | 0.409 |  |  |  |
| Jola | 8 (10.8) | 70 (9.9) | 1.20 (0.54; 2.69) | 0.655 |  |  |  |
| Serere | 3 (4.1) | 34 (4.8) | 0.93 (0.27; 3.17) | 0.905 |  |  |  |
| Serahula | 2 (2.7) | 20 (2.8) | 1.05 (0.24; 4.67) | 0.948 |  |  |  |
| Manjago | 1 (2.7) | 9 (1.3) | 2.34 (0.49; 11.22) | 0.289 |  |  |  |
| Other | 1 (1.4) | 26 (3.7) | 0.40 (0.05; 3.07) | 0.381 |  |  |  |
| **Number of persons sleeping in one bedroom,** N=783 | n=74 | n=709 |  |  |  |  |  |
| 6 or less | 71 (96.0) | 688 (97.0) | 1 | -- | 1 | 1 | -- |
| 7 or more | 3 (4.0) | 10 (1.4) | 2.91 (0.78; 10.8) | 0.095 | 3.53 (0.89; 13.98) | 4.25 (0.94;19.08)* | 0.072; 0.059* |
| Unknown | 0 (0.0) | 11 (1.6) | 0 | -- | 0 | 0 | -- |
| **Duration of education of the mother**, N=783 | n=74 | n=709 |  |  |  |  |  |
| 0-4 years | 25 (33.8) | 174 (24.5) | 1.79 (1.01; 3.13) | 0.042 | 1.94 (1.10; 3.44) | 2.31 (1.25; 4.26)* | 0.023; 0.007* |
| 5 and more years | 30 (40.5) | 373 (52.6) | 1 | -- | 1 | 1 | -- |
| Unknown | 19 (25.7) | 162 (22.9) | 1.46 (0.80; 2.67) | 0.221 | 1.40 (0.75; 2.60) | 1.21 (0.63; 2.34)* | 0.290; 0.567* |
| Mean duration of education of the mother, N=602 | n=55 | n=547 |  |  |  |  |  |
| Mean (95% CI) | 5.8 (4.88-6.79) | 6.4 (6.10-6.65) |  | 0.126 |  |  |  |
| **Education of the mother**, N=780 | n=74 | n=706 |  |  |  |  |  |
| None | 11 (14.9) | 92 (13.0) | 1.05 (0.51; 2.15) | 0.896 |  |  |  |
| Madrassa | 35 (47.3) | 307 (43.5) | 1 | -- |  |  |  |
| Primary school | 9 (12.2) | 117 (16.6) | 0.67 (0.31; 1.45) | 0.312 |  |  |  |
| Secondary school | 19 (25.7) | 190 (26.9) | 0.88 (0.49; 1.58) | 0.662 |  |  |  |
| **Education of the father**, N=783 | n=74 | n=709 |  |  |  |  |  |
| None | 3 (4.1) | 47 (6.6) | 0.45 (0.13; 1.53) | 0.204 | 0.49 (0.14; 1.67) | 0.50 (0.14; 1.78)* | 0.254; 0.285* |
| Madrassa | 36 (48.5) | 256 (36.1) | 1 | -- | 1 | 1 | -- |
| Primary school | 3 (4.1) | 39 (5.5) | 0.55 (0.16; 1.86) | 0.334 | 0.65 (0.19; 2.24) | 0.72 (0.20; 2.63)* | 0.491; 0.617* |
| Secondary school | 29 (39.2) | 343 (48.4) | 0.60 (0.36; 1.00) | 0.053 | 0.58 (0.34; 0.99) | 0.61 (0.34; 1.08)* | 0.046; 0.088* |
| Unknown | 3 (4.1) | 24 (3.4) | 0.89 (0.25; 3.10) | 0.853 | 0.82 (0.23; 2.89) | 0.81 (0.21; 3.10)* | 0.760; 0.761* |
| **Use of bednets,** N=781 | n=74 | n=707 |  |  |  |  |  |
| Never | 41 (55.4) | 390 (55.2) | 1 | -- |  |  |  |
| Sometimes | 107 (16.2) | 95 (13.4) | 1.11 (0.64; 1.93) | 0.707 |  |  |  |
| Always | 74 (28.4) | 222 (31.4) | 1.34 (063; 2.82) | 0.449 |  |  |  |
| **Impregnation of bednets,** N=346 | n=33 | n=313 |  |  |  |  |  |
| Never | 25 (75.8) | 198 (63.3) | 1 | -- |  |  |  |
| More than 12 months ago | 1 (3.0) | 31 (9.9) | 0.44 (0.05; 4.15) | 0.476 |  |  |  |
| 6-12 months ago | 4 (12.1) | 55 (17.6) | 1.42 (0.29; 6.79) | 0.659 |  |  |  |
| Less than 6 months ago | 3 (9.0) | 29 ( 9.3) | 1.74 (0.58; 5.20) | 0.324 |  |  |  |
| **Last malaria treatment**, N=783 | n=74 | n=709 |  |  |  |  |  |
| More than 9 months ago | 38 (53.5) | 362 (55.5) | 1 | -- |  |  |  |
| 3-9 months ago | 25 (35.2) | 213 (32.7) | 1.12 (0.66; 1.90) | 0.681 |  |  |  |
| 1-3 months ago | 4 (5.6) | 52 (8.0) | 0.73 (0.25; 2.14) | 0.569 |  |  |  |
| Less than 1 months ago | 4 (5.6) | 25 (3.8) | 1.52 (0.50; 4.61) | 0.456 |  |  |  |
| Unknown | 3 (4.0) | 57 (8.1) | 0.50 (0.15; 1.68) | 0.263 |  |  |  |

Values presented in the table represent “number (%)” if not stated otherwise.

* Adjusted OR and p values for first or more pregnancies, if baby was born in PM season, year of birth, and duration of schooling of the mother

**In 2005 study participants were recruited from January till July only. Trend is significant for 2002-04 as well p<0.0001

*** OR comparing odds for January-July 2005 with odds for January-July 2002
