# Supplementary material for: Placental Malaria is associated with reduced early life weight development of affected children independent of low birth weight
Source: Malar J. 2010 Jan 14;9:16. doi: 10.1186/1475-2875-9-16 (PMC2841609; doi:10.1186/1475-2875-9-16)
Supplement: Additional file 2 — Association of placental malaria with maternal anaemia and baby's characteristics at birth. The table in this file shows the results of the statistical analysis investigating if maternal and baby's outcome characteristics at birth were different between PM+ and PM- groups. [file 1475-2875-9-16-S2.DOC]

**Additional file 2:**

**Association of placental malaria infection with maternal anaemia and baby’s characteristics at birth**

|  | **Univariable analysis** | | | | **Multivariable analysis** | | |
| --- | --- | --- | --- | --- | --- | --- | --- |
|  | **2002-05** | | | | **2002-04** | | |
|  | **Infected** | **Not infected** | OR (95%CI) | p-value | OR (95%CI) | | p-value; adjusted p-value* |
|  |  |  |  |  | Crude | Adjusted* |  |
| **Maternal anaemia**, N=783 | *n=74* | *n=709* |  | 0.866 |  |  |  |
| Normal (Hb≥11.0) | 27 (42.2) | 247 (34.8) | 1 | -- |  |  |  |
| Mild anaemia (10.0≤ Hb <11.0) | 20 (21.3) | 203 (28.6) | 0.9 (0.49; 1.65) | 0.737 |  |  |  |
| Moderate anaemia (7.0≤ Hb<10.0) | 17 (26.6) | 182 (25.7) | 0.85 (0.45; 1.61) | 0.628 |  |  |  |
| Severe anaemia (Hb<7.0) | 0 | 0 | -- | -- |  |  |  |
| Unknown | 10 (13.5) | 77 (10.9) | 1.19 (0.55; 2.56) | 0.661 |  |  |  |
| **Child moratility**, N=783 | *n=74* | *n=709* |  |  |  |  |  |
| Child alive | 71 (96.0) | 694 (97.9) | 1 | -- |  |  |  |
| Child died | 3 (4.1) | 15 (2.1) | 1.95 (0.55; 6.91) | 0.298 |  |  |  |
| **Sex of the baby**, N=783 | *n=74* | *n=709* |  |  |  |  |  |
| Male | 39 (52.7) | 385 (54.3) | 1 | -- |  |  |  |
| Female | 35 (47.3) | 324 (45.7) | 1.07 (0.66; 1.72) | 0.793 |  |  |  |
| **Weight of the baby**, N=783 | *n=74* | *n=709* |  | 0.337 |  |  |  |
| Low birth weight (<2,5kg) | 11 (14.9) | 69 (9.7) | 1.64 (0.82; 3.26) | 0.160 | 1.84 (0.91; 3.72) | 1.46 (0.67; 3.17)* | 0.088; 0.336* |
| Normal birth weight (>= 2,5kg) | 62 (83.7) | 637 (89.8) | 1 | -- | 1 | 1 | -- |
| Unknown | 1 (1.4) | 3 (0.5) | 3.42 (0.35; 33.42) | 0.290 |  |  |  |
| Mean  (95% CI) | 2,972  (2,860; 3,082) | 3,052  (3,019; 3,085) |  | 0.139 |  |  |  |
| **Length of the baby** [cm], N=777 | *n=73* | *n=704* |  |  |  |  |  |
| Mean (95% CI) | 48.2 (47.6; 48.7) | 48.6 (48.4; 48.7) |  | 0.128 |  |  |  |

* Adjusted OR and p values for first or more pregnancies, if baby was born in PM season, year of birth, and duration of schooling of the mother.
